# The risk of fracture and prevalence of osteoporosis is elevated in patients with idiopathic inflammatory myopathies: cross-sectional study from a single Hungarian center

**DOI:** 10.1186/s12891-020-03448-2

**Published:** 2020-07-02

**Authors:** Anett Vincze, Levente Bodoki, Katalin Szabó, Melinda Nagy-Vincze, Orsolya Szalmás, József Varga, Katalin Dankó, János Gaál, Zoltán Griger

**Affiliations:** 1 Division of Clinical Immunology, Faculty of Medicine, University of Debrecen, Móricz Zsigmond út 22, Debrecen, H-4032 Hungary; 2 Gyula Petrányi Doctoral School of Clinical Immunology and Allergology, University of Debrecen, Debrecen, Hungary; 3 Division of Rheumatology, Faculty of Medicine, University of Debrecen, Debrecen, Hungary; 4 Department of Medical Imaging, Faculty of Medicine, University of Debrecen, Debrecen, Hungary; 5 Department of Medical Imaging, Division of Nuclear Medicine, University of Debrecen, Debrecen, Hungary; 6 Department of Medicine, Kenézy Gyula University Hospital, University of Debrecen, Debrecen, Hungary

**Keywords:** Fracture risk, Vertebral fractures, Myositis, Rheumatoid arthritis

## Abstract

**Background:**

The prevalence of osteoporosis and risk of fractures is elevated in rheumatoid arthritis (RA), but we have limited information about the bone mineral density (BMD) and fracture risk in patients with inflammatory myopathies. We intended to ascertain and compare fracture risk, bone mineral density and the prevalence of vertebral fractures in patients with inflammatory myositis and rheumatoid arthritis and to assess the effect of prevalent fractures on the quality of life and functional capacity.

**Methods:**

Fifty-two patients with myositis and 43 patients with rheumatoid arthritis were included in the study. Fracture Risk was determined using FRAX® Calculation Tool developed by the University of Sheffield. Dual energy X-ray absorptiometry and bidirectional thoracolumbar radiographs were performed to assess BMD and vertebral fractures. Quality of life was measured with Short Form-36 (SF-36) and physical function assessment was performed using Health Assessment Questionnaire (HAQ).

**Results:**

We found a significantly elevated fracture risk in RA as compared to myositis patients if the risk assessment was performed without the inclusion of the BMD results. If BMD results and glucocorticoid dose adjustment were taken into account, the differences in fracture risk were no longer significant. The prevalence of osteoporosis was found to be significantly higher in the myositis group (7% vs. 13.5%, p: 0.045), but the fracture prevalence was similar in the two groups (75% vs. 68%). The fracture rates were independently associated with age in the myositis group, and with lower BMD results in the RA patients. The number of prevalent fractures was significantly correlated to poorer physical function in both groups, and poorer health status in the myositis group, but not in the RA group.

**Conclusions:**

Our findings suggest that inflammatory myopathies carry significantly elevated risks for osteoporosis and fractures. These higher risks are comparable to ones detected with RA in studies and strongly affect the physical function and quality of life of patients. Therefore further efforts are required to make the fracture risk assessment reliable and to facilitate the use of early preventive treatments.

## Background

Osteoporosis is a common metabolic skeletal disorder characterized by decreased bone mass and deteriorated bone structure, leading to increased fracture rate [1]. Since the average age and the proportion of elderly persons in the population is increasing continuously, osteoporosis and consecutive fractures have become a global public health problem with enormous socioeconomic consequences [2, 3]. It is widely known that rheumatoid arthritis (RA) is one of the most important causes of secondary osteoporosis. Osteoporotic bone fractures are of crucial importance in the functioning and quality of life of patients. The pathogenesis of bone loss in autoimmune disorders is multifactorial. The increased serum and tissue levels of pro-inflammatory mediators lead to the increased expressions of receptor activator of nuclear factor kappa-Β ligand (RANKL) by osteoblasts, T-lymphocytes and synovial fibroblasts. The RANKL-RANK binding is the main pathogenetic event of osteoclastogenesis, osteoclast maturation and functioning. Another, not at all negligible reason, is the synergism of several factors that negatively affect the bone mass: dietary factors (decreased calcium /Ca/ and vitamin D3 intake), decreased muscle mass/strength and functional capacity, immobilization, deficient intestinal Ca absorption, reduced levels of sexual steroids, avoidance of sunlight and use of sunscreens and, last but not least, glucocorticoid (GC) use [4–10]. The chronic GC exposure leads to decreased calcium absorption, increased renal Ca loss, secondary hyperparathyroidism, decreased sexual hormone levels, decreased number and function of osteoblasts and eventually increased bone resorption and reduced bone formation [11, 12]. In patients with RA, systemic osteoporosis coincides with local bone resorption, as a typical consequence of inflammatory synovitis. Vertebral fractures are important but yet under recognized manifestations of osteoporosis. Most of them are asymptomatic, which makes their recognition more difficult, consequently, they might remain unnoticed for years. Clinical observations show that 30% of patients taking steroids for more than 3 years suffer from an osteoporotic fracture. Moreover, literature data also demonstrated that patients with polymyositis (PM) or dermatomyositis (DM) had significantly lower BMD in both the hip and lumbar (L) spine compared to the healthy, age- and gender-matched population [10, 13]. The Fracture Risk Assessment Tool (FRAX®) developed and validated by Kanis et al., more than 10 years ago, is still the most widely accepted and used method in clinical practice to estimate the 10-year probability of osteoporotic fractures [14]. FRAX score takes into account the relevant risk factors for a bone fracture, e.g., the presence of RA, but not myositis.

Our present work is a cross-sectional observational study, in which we intended to answer the following questions: 1. What is the prevalence of low BMD, vertebral fracture and high fracture risk in our patients with inflammatory myositis and RA? 2. Which factors are associated with higher fracture rates in myositis and RA patients? 3. How do the vertebral fractures influence the physical function and quality of life of patients?

## Methods

This scientific cross-sectional study was conducted on our own initiative, in 52 consecutive patients with myositis and 43 patients with RA under the care of the National Myositis Center, in the Division of Clinical Immunology, Faculty of Medicine, at the University of Debrecen between January 2017–June 2018. This study meets, and is in compliance with all ethical standards of medicine. Informed consent was obtained from all of the subjects. This study is ethically compliant and was carried out in compliance with the Declaration of Helsinki. The eligibility criteria were the diagnosis of probable, or definitive idiopathic inflammatory myopathy (IIM) based on the Bohan and Peter criteria [15], and rheumatoid arthritis according to the 2010 American College of Rheumatology-European League Against Rheumatism (ACR-EULAR) classification criteria [16]. The patients with confounders of bone health were excluded from the study: if the patient took any drug affecting bone mineral density (including bisphosphonates, thiazide diuretics, anticoagulants, anticonvulsants, glitazones, etc.) except for vitamin D3 and Ca, but including secondary osteoporosis and those patients suffering from malignancies. In total, 121 patients were included at the start of the study, and finally 26 individuals were excluded based on the presence of exclusion criteria, or missing BMD and/or FRAX data.

Laboratory tests included the measurements of calcium, alkaline phosphatase, C-reactive protein (CRP), thyroid-stimulating hormone, serum 25-OH Vitamin D3 levels and bone turnover markers (BTM): (parathyroid hormone, osteocalcin /OC/, beta-crosslaps, C-terminal telopeptides of type-I collagen /CTX-I/). Blood sampling was done after overnight fasting to measure levels of PTH, OC and CTX-I. Plasma 25-OH-D3 level was analyzed by high pressure liquid chromatography (HPLC) using a Jasco HPLC system (Jasco, Tokyo, Japan) and Bio-Rad reagent kit (Bio-Rad Laboratories, Hercules, CA, USA). Serum PTH, OC and CTX-I were measured using electrochemiluminescence immunoassay (Roche Diagnostics GmbH, Mannheim, Germany). The inter-assay CV was < 7% for PTH (lower detection limit: 0.127 pmol/L, upper detection limit: 530 pmol/L), < 4% for OC (lower detection limit: 0.5 μg/L, upper detection limit: 300 μg/L) and < 7% for CTX-I (lower detection limit: 0.010 μg/L, upper detection limit: 6 μg/L).

We measured the BMD of the lumbar spine (L_1–4_ vertebrae) and the left femoral neck by AP-DXA. The scan was performed with a DPX Pro bone densitometer (GE-Lunar Radiation Corporation, Madison, WI, USA), according to the manufacturer’s protocol. In patients with a history of a previous hip fracture, hip replacement surgery, or severe joint destruction, we measured bone mineral density in the right femoral neck. Osteoporosis was diagnosed according to the criteria proposed by the World Health Organization Study Group, when the BMD was 2.5 or more standard deviations below the young-adult mean, and osteopenia was diagnosed when the BMD was between − 1 and − 2.5 [17].

The FRAX, Health Assessment Questionnaire (HAQ) and Short Form 36 (SF-36) questionnaires were completed by a personal interviewer. The web-based algorithm at http://www.shef.ac.uk/FRAX® was applied as the FRAX® algorithm (version 3.6) adapted for Hungary [18]. Special risk factors (age, sex, weight, height, previous fracture, parental hip fracture, current smoking, GCs, RA, secondary osteoporosis, alcohol 3 or more units/day, femoral neck BMD) were recorded into this calculator, for every single patient. The output was a 10-year probability of hip fracture and a 10-year probability of a major osteoporotic fracture (clinical spine, forearm, hip or shoulder fracture) [18]. We measured quality of life including mental health with the SF-36 questionnaire validated for use in Hungary [19]. The assessment of the patients’ physical function was performed using the HAQ questionnaire [20].

To assess the prevalence of vertebral fractures patients underwent a bidirectional (anteroposterior and lateral) X-ray imaging of the thoracic (Th) and lumbar (L) spine on separate cassettes for each picture. To decrease probability of potential selection bias, all of the patients were individually instructed to undergo X-ray examinations, on personally scheduled dates, and finally 40 myositis and 35 RA patients were able to complete the study. On standard radiographs the Genant’s semi-quantitative assessment was used to evaluate vertebral fractures [21]. Vertebral shape (wedge, concave, or crush) and decreases in anterior, posterior, and/or middle vertebral height (grade 0: no reduction; grade 1: minimal fracture, 20–25% height decrease; grade 2: moderate fracture, 25–40% height decrease; and grade 3: severe fracture, greater than 40% height decrease) were determined by two independent assessors (A.V. and O.S.). Their differences were resolved by repeated analysis and by consensus.

Statistical analysis was performed with version 26 of the SPSS software package (IBM Corp., Armonk, NY, USA). The normality of the distributions in case of continuous variables was tested using the Shapiro-Wilk test. Normally distributed continuous variables were described by mean and standard deviation values (SD). Categorical variables were described using frequencies (case number) and percentages. For comparing the groups we used independent samples t-test, or Mann-Whitney test depending on the distribution. The connection of two scalar variables was characterized by Spearman’s correlation, while in case of binary variables we used Fisher’s exact test. To identify the risk factors of vertebral fracture, we applied stepwise discriminant analysis (Wilks), also. The multivariate general linear model was applied to determine the factors that influence HAQ and SF36 results. *P* values of less than 0.05 were regarded as statistically significant.

## Results

One hundred and twenty-one Caucasian patients participated in the study and fullfilled the inclusion criteria, while 26 patients were excluded based on the presence of exclusion criteria, or missing BMD, or FRAX results. The final myositis group consisted of 52 patients (9 males and 43 females, with a mean age of 57.46 years), while the RA group consisted of 43 patients (2 males and 41 females, with a mean age of 58.58 years). There were no significant differences between the two groups in the basic clinical data (including the mean BMD and 25 OH Vitamin D3 level) as indicated in Table 1. The proportion of patients receiving oral Ca and vitamin D substitution did not differ significantly between the two groups (34 vs. 29 patients). We could not find any significant differences between the two groups in terms of other factors included in the FRAX® tool (previous fracture, parental hip fracture, smoking, glucocorticoids, alcohol consumption) except the presence of rheumatoid arthritis (data not shown). In the myositis and RA groups normal BMD was found in 27 and 53.5%, respectively, whilst osteopenia was found in 60 and 39.5% of the patients, respectively, and osteoporosis found in 13.5 and 7% of the patients, respectively, and the difference in frequency of osteoporosis found to be statistically significant between the two groups (*Fisher’s exact tes*t, *p* = 0.045).

**Table 1:** Summary of the most relevant clinical data and FRAX scores of the patients

	Myositis *N* = 52		Rheumatoid arthritis *N* = 43		*P*-value (95% CIoD)
	Mean^a^/Median^b^	SD^a^/Min-Max^b^	Mean^a^/Median^b^	SD^a^/Min-Max^b^	
Age (years)^a^	57.46	11.168	58.58	10.486	0.618 (−5.6–3.3)
Men (N) Women (N)	9 (17.3%) 43 (82.7%)		2 (4.7%) 41 (95.3%)		0.104
Menopause at examination (N)	33/43 (76.67%)		35/41% (85.3%)		0.314
Weight (kg)^a^	70.88	14.38	73.74	13.77	0.328 (−2.9–2.9)
Height (cm) ^a^	164.12	7.56	163.7	7.1	0.308 (0.4–1.5)
BMI (kg/m^2^) ^a^	26.39	5.58	27.5	4.6	0.318 (−1.1–1.1)
Vitamin D and calcium supplement (N)	34 (65.4%)		29 (67.4%)		0,833
25 OH Vitamin D3 level (nmol/L) ^b^	59.5	15.2–125.2	62.5	27.5–129.2	0.196
BMD L1–4 (g/cm^2^)^a^	1.04	0,238	1.07	0.159	0.557 (−0.08–0.07)
BMD femur (g/cm^2^) ^a^	0.83	0,108	0.85	0.125	0.294 (−0.09–0.01)
**Normal (N)**	**14 (27%)**		**23 (53.5%)**		**0.045**
**Osteopenia (N)**	**31 (60%)**		**17 (39.5%)**		
**Osteoporosis (N)**	**7 (13.5%)**		**3 (7%)**		
*FRAX score: MOF*					
**Without DEXA**^**1a**^	**9.68%**	**7.42**	**15.58%**	**10.91**	**0.008**
With DEXA^2a^	9.44%	6.723	13.25%	9.43	0.053
Steroid dose adjusted with DEXA^3a^	9.54%	7.475	9.96%	7.968	0.884
*FRAX score: HF*					
**Without DEXA**^**1a**^	**3.06%**	**3.97**	**6.23%**	**7.20**	**0.022**
With DEXA^2a^	2.77%	3.01	3.57%	5.08	0.811
Steroid dose adjusted with DEXA^3a^	2.87%	3.393	2.46%	3.382	0.128

*BMI* body mass index, *BMD L1–4* Bone Mineral Density from lumbal 1–4 vertebrae, *BMD femur* Bone Mineral Density in the left femoral neck, Normal-Osteopenia-Osteoporosis: the condition of the bone according to the T-score result, *FRAX* Fracture Risk Assessment Tool, *MOF* Major Osteoporotic Fracture, *HF* Hip Fracture, *DEXA* dual-energy x-ray absorptiometry

Significances were calculated with independent samples t-test or Mann-Whitney test, according to the distribution. Normality of the distributions was checked using Shapiro-Wilk test. Data are presented as mean and standard deviation (SD) with normal distribution (a on the upper corner of the variable) and median, minimum, maximum with non-gaussian distribution (b on the upper corner of the variable); 95% CIoD: 95% Confidence Interval of the Difference (lower-upper). Categorical variables were described using frequency (case number) and percentage. FRAX scores were calculated 1: without the DEXA levels and without steroid dose adjustment (steroid yes/no only); 2: With DEXA levels without steroid dose adjustment (steroid yes/no only); 3: With DEXA levels and steroid dose adjustment

The fracture risk assessment was calculated first without applying the BMD values. Regarding the other major and femoral neck fractures the fracture risk in RA patients was significantly higher than in myositis patients (15.58% vs. 9.68 and 6.23% vs 3.06%; *p* = 0.008 and *p* = 0.022). As a second step, the fracture risk calculation was repeated, and this time with the BMD values taken into account, with the earlier significant difference in the fracture probability disappearing (13.25% vs. 9.44 and 3.57% vs. 2.77%; *p* = 0.053 and *p* = 0.811). During the third step, the fracture risk assessment was performed after adjustment to the dose of glucocorticoids according to Kanis et al. [22]. With this correction the magnitude of the difference further decreased: the risk of major osteoporotic and hip fractures were found to be 9.96% vs 9.54% (*p* = 0.884) and 2.46% vs. 2.87% (*p* = 0.128) (Table 1).

As previously mentioned 75 patients underwent bidirectional vertebral X-ray examinations, 40 myositis patients (8 males and 32 females, mean age 60.97 years) and 35 RA patients (all female, mean age 59.71 years). Patients with myositis had significantly longer disease duration (13 vs. 7 years, *p* = 0.021) and higher cumulative steroid dose (17.6 g vs. 4.1 g, *p* = 0.009) (Table 2). Overall 194 vertebral fractures were discovered in 54 patients (115 fractures in 30 myositis and 79 fractures in 24 RA patients), with these patients representing 75% of the myositis group and 68% the RA group, and the difference was not statistically significant (Table 2). As a next step the myositis and RA patients were divided into two groups according to the presence of vertebral fractures. The mean age of the fractured patients was significantly higher in both groups using Mann-Whitney test (62.83 vs. 55.4; *p* = 0.034 in the myositis group, and 63.25 vs. 52,0; *p* = 0.022 in the RA group; Table 3), which was similarly found with stepwise discriminant analysis in myositis (*p* = 0.042), but age was not an independent predictor of vertebral fractures in the RA group. In addition significantly lower lumbar and femur neck BMD were seen in fractured patients in the RA group (1.0 g/cm2 vs 1.19 g/cm and 0.83 g/cm2 vs 0.94 g/cm2; *p* = 0.008 and *p* = 0.01. The correlation of lower lumbar BMD and fractures in RA was further confirmed by stepwise discriminant analysis (*p* = 0.001), but with this method femur-neck BMD and age of the RA patients were not profound independent significant factors, according to their interdependency. The mean 25-OH Vitamin D3 levels showed no correlation with the presence of vertebral fractures (Table 3).

**Table 2:** Basic clinical data of patients available for vertebral X-ray assessments

	Myositis *N* = 40		Rheumatoid arthritis *N* = 35		*P*-value
Age (years) ^a^	60.97	10.09	59.71	11.16	0.795
Female/male (N)	32/8		35/0		–
**Duration of disease (years)**^b^	**13**	**1–28**	**7**	**1–29**	**0.021**
**Cumulative steroid dose (g)**^b^	**17.6**	**0–13.5**	**4.1**	**0–55**	**0.009**
Patients with vertebral fractures (N)	30		24		0.375
Number of all fractures (N)	115		79		0.206

Significances were calculated with independent samples t-test or Mann-Whitney test, according to the distribution. Normality of the distributions was checked using Shapiro-Wilk test. Data are presented as mean and standard deviation (SD) with normal distribution (a on the upper corner of the variable) and median, minimum, maximum with non-gaussian distribution (b on the upper corner of the variable); 95% CIoD: 95% Confidence Interval of the Difference (lower-upper). Categorical variables were described using frequency (case number) and percentage

**Table 3:** Clinical and laboratory data of myositis and RA patients with and without fractures

*Characteristics of myositis patients*	With fracture *N* = 30		Without fracture *N* = 10		*P*-value
	Mean^a^/Median^b^	SD^a^/Min-Max^b^	Mean^a^/Median^b^	SD^a^/Min-Max^b^	
**Age (years)**^**a**^	**62.83**	**9.858**	**55.4**	**9.057**	**0.034**
Duration (years)^b^	13.5	1–28	9.5	1–21	0.16
Cumulative steroid (g)^b^	20.2	0–135.4	14.6	0.2–71.3	0.79
BMD L1–4 (g/cm^2^) ^b^	1.08	0.79–1.54	1.04	0.84–1.15	0.20
BMD femur (g/cm^2^) ^b^	0.82	0.62–1.05	0.84	0.67–1.02	0.76
25OH-Vitamin D3 level (nmol/L)^b^	57.4	27.8–125.2	66.2	24.2–90	0.61
ß-CTx (ug/L) ^b^	0.28	0.05–0.81	0.21	0.1–0.46	0.43
*Characteristics of RA patients*	With fracture *N* = 24		Without fracture *N* = 11		*P*-value
**Age (years)**^**a**^	**63.25**	**9.175**	**52**	**11.610**	**0.022**
Duration (years) ^b^	6.5	1–29	7	1–26	0.97
Cumulative steroid (g)^b^	4	0–55	4.3	0–28	0.430
**BMD L1–4 (g/cm**^**2**^**)**^**b**^	**1.00**	**0.85–1.42**	**1.19**	**1–1.41**	**0.008**
**BMD femur (g/cm**^**2**^**)**^**b**^	**0.83**	**0.7–1.02**	**0.94**	**0.84–1.11**	**0.010**
25OH-Vitamin D3 level (nmol/L) ^b^	69.0	29.2–129.2	53.2	27.5–85.5	0.11
ß-CTx (ug/L) ^b^	0.26	0.07–0.57	0.22	0.07–0.45	0.3

*Duration* of the disease in years, *BMD L1–4 B*one Mineral Density from the lumbar 1–4 vertebrae, *BMD femur* Bone Mineral Density in the left femoral neck, *ß-CTx* beta-crosslaps

Significances were calculated with independent samples t-test or Mann-Whitney test, according to the distribution. Normality of the distributions was checked using Shapiro-Wilk test. Data are presented as mean and standard deviation (SD) with normal distribution (a on the upper corner of the variable) and median, minimum, maximum with non-gaussian distribution (b on the upper corner of the variable). Stepwise discriminant analysis (Wilks) was also performed, data are found in the text

**Fig. 1:**
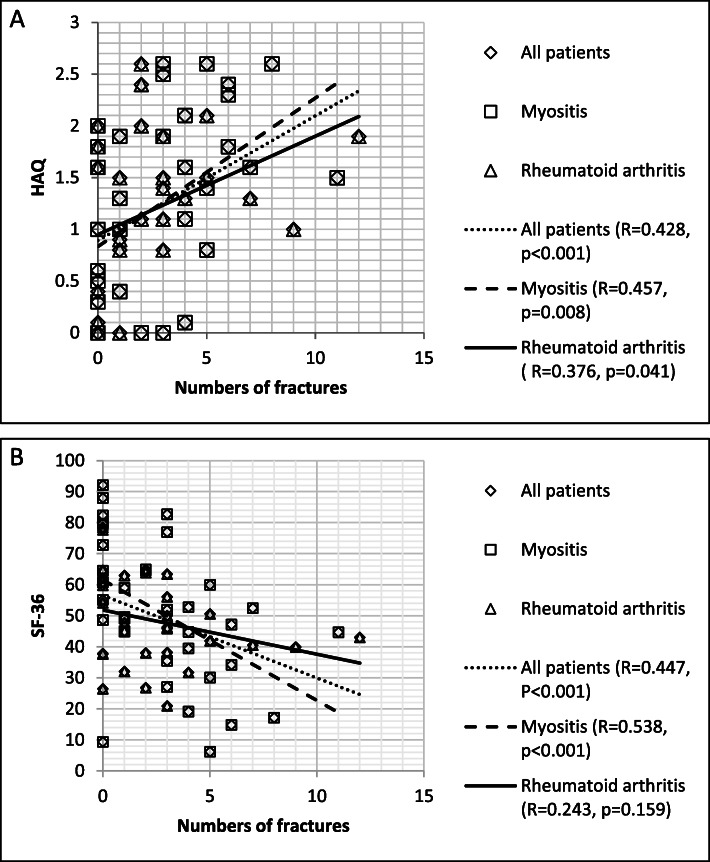
**a** The effect of the number of bone fractures on the physical function (HAQ) and **b** quality of life (SF-36) in myositis, rheumatoid arthritis, and in all patients. HAQ: Health Assessment Questionnaire, SF-36: Short Form-36.Regression analysis (Data of multivariate general linear model analysis are presented in the text)

Finally we investigated the influence of vertebral fractures on these patients’ physical function and quality of life using HAQ and SF-36 questionnaires (Fig. 1a-b). It was found that the decrease in physical function and quality of life was proportional to the number of vertebral fractures if we analyzed the two groups together. In addition female gender was significantly associated with poor SF-36 results (*p* = 0.015). The worsening of physical function was more pronounced in the myositis group compared to the RA group (R = 0.457; *p* = 0.008 vs. R = 0.376; *p* = 0.041). (Fig. 1a - Correlation between number of fractures and the results of the functional tests /HAQ/). Surprisingly, we could not detect any significant correlations with regard to the SF-36 data of patients with RA, but in myositis patients and in the total patient group the number of bone fractures was strongly associated with poor SF-36 results (Fig. 1b - Correlation between number of fractures and the results of the patient’s health /SF-36/). Furthermore, general linear model analysis showed that in the RA group the history of previous fractures (*p* = 0.024), and also its co-occurrence with steroid treatment (*p* = 0.032) were significant factors for poor SF-36 results (data not shown). The same results were found, if we examined separately the mental and the physical components of the questionnaire (data not shown).

## Discussion

To our knowledge this is the first study, which investigates and compares bone fracture risks in IIM and RA, and also the first work that correlates BMD, FRAX and vertebral fracture data of myositis patients with rheumatoid arthritis patients. Data from a recent population based study in Taiwan also showed a higher osteoporosis prevalence rate among patients with DM/PM. The increased osteoporosis risk was independent of the corticosteroid and immunosuppressant treatments [9]. Gupta et al. recently published a study about prevalence of vertebral deformities in patients with inflammatory myositis and found a high prevalence of asymptomatic vertebral fractures, but fracture risk and the consequences of fracture on physical function and quality of life were not examined [23]. Basically, fracture risk assessed without taking BMD into consideration showed a greater risk of fracture in patients with rheumatoid arthritis than in myositis patients. If the BMD data were applied as well, it showed there was no longer any significant differences between the values of the two groups. This might support the argument for the lower BMD – which is more frequent in patients with myositis - counterbalances the “confounding” effect of RA as a risk factor in the FRAX tool. With an adjustment in FRAX according to the dose of glucocorticoids, the remaining non-significant differences were further decreased. Taking into account the high prevalence of osteoporosis/osteopenia in the myositis group, it seems logical to consider incorporating a factor that modifies the FRAX tool and allows for a more reliable risk calculation in patients with myositis. Of course, this requires studies with a larger patient population and with bone fracture endpoints. In addition, it would generate a necessity for multiple, disease dependent modifying factor development according to other systemic musculoskeletal diseases (lupus, Sjögren’s syndrome, vasculitis, etc). We showed that the fractured patients were significantly older in myositis, but had lower lumbar BMD levels in RA. The occurrence of vertebral fractures in both myositis and rheumatoid arthritis were very common and seriously affected the patients’ physical function and quality of life, especially in those with multiple fractures. It is interesting to observe that this effect was more pronounced in females and in myositis patients with regard to the HAQ results, and, surprisingly, the fractures did not significantly modify the health status of the RA patients. This latter phenomenon could be explained by the frequent joint damage and secondary fibromyalgia seen in RA, which might bias the results of the questionnaire. We found a similarly high prevalence rate of vertebral fracture as Gupta et al. [22], but in their myositis population the median age and disease duration were shorter than in our population, and only patients with myositis were investigated. Despite the longer duration of the disease in our population the prevalence of fractures was not more frequent, therefore it is logical to speculate that the majority of fractures occur in the early phase of the disease, when the administration of higher corticosteroid doses is more frequent. Based on the results of our study, a national patient educational material, and a patient advisory card has been constructed, with an aim to increasing the patients’ awareness and adherence to preventive pharmacological and non-pharmacological antiporotic treatments.

The possible limitations of this study should be acknowledged. This work was a single center study from a national myositis unit in Hungary, and the number of participants in the study was relatively low. The lower number of patients with vertebral X-ray examinations could be a cause for selection bias, and due to the cross sectional nature of the investigation the calculated and the real fracture risks were not comparable.

## Conclusions

It can be concluded that osteoporosis and consequential fractures in myositis are common and probably underestimated, and that their examination is often neglected. Therefore, it would be important to pay greater attention to the recognition of low BMD and high fracture risk and adherence to preventive measures. Our results showed a good concordance with data of groups from other regions of the world, suggesting that the high fracture prevalence is a global myositis dependent feature. Beyond that, in our opinion, the use of validated, internationally accepted patient warning cards could increase patients’ compliance and will lead to decreased fracture rates. We believe, that through the collaboration of myositis centers we can integrate myositis into the FRAX calculator as a unique risk factor. This way we can make the risk assessment more reliable worldwide in patients with inflammatory myositis.

## Data Availability

The datasets used and/or analysed during the current study are available from the corresponding author on reasonable request.

## Abbreviations

ACR: American College of Rheumatology
ß-CTx: Beta-crosslaps
BMD: Bone mineral density
BMI: Body mass index
BTM: Bone turnover markers
Ca: Calcium
CRP: C-reactive protein
CTX-I: C-terminal telopeptides of type-I collagen
DEXA: Dual-energy x-ray absorptiometry
DM: Dermatomyositis
EULAR: European League Against Rheumatism
FRAX: Fracture Risk Assessment Tool
GC: Glucocorticoid
HAQ: Health Assessment Questionnaire
HPLC: High pressure liquid chromatography
IIM: Idiopathic inflammatory myopathy
L: Lumbar
OC: Osteocalcin
PM: Polymyositis
PTH: Parathyroid hormone
RA: Rheumatoid arthritis
RANK: Receptor activator of nuclear factor kappa-Β
RANKL: Receptor activator of nuclear factor kappa-Β ligand
SD: Standard deviation
SF-36: Short Form-36
TNF-α: Tumor necrosis factor alpha
ug: Microgram

## References

[1] Kanis JA, Melton LJ, Christiansen C, Johnston CC, Khaltaev N (1994). The diagnosis of osteoporosis. J Bone Miner Res.

[2] Bliuc D, Nguyen ND, Nguyen TV, Eisman JA, Center JR (2013). Compound risk of high mortality following osteoporotic fracture and refracture in elderly women and men. J Bone Miner Res.

[3] Frost SA, Nguyen ND, Center JR, Eisman JA, Nguyen TV (2013). Excess mortality attributable to hip-fracture: a relative survival analysis. Bone.

[4] Lacativa PG, Farias ML (2010). Osteoporosis and inflammation. Arq Bras Endocrinol Metabol.

[5] Rehman Q, Lane NE (2001). Bone loss. Therapeutic approaches for preventing bone loss in inflammatory arthritis. Arthritis Res.

[6] Santiago RA, Silva CA, Caparbo VF, Sallum AM, Pereira RM (2008). Bone mineral apparent density in juvenile dermatomyositis: the role of lean body mass and glucocorticoid use. Scand J Rheumatol.

[7] van Staa TP, Leufkens HG, Cooper C (2002). The epidemiology of corticosteroid-induced osteoporosis: a meta-analysis. Osteoporos Int.

[8] Mazess RB, Whedon GD (1983). Immobilization and bone. Calcif Tissue Int.

[9] Lee CW, Muo CH, Liang JA, Sung FC, Hsu CY, Kao CH (2016). Increased osteoporosis risk in dermatomyositis or polymyositis independent of the treatments: a population-based cohort study with propensity score. Endocrine.

[10] LeBlanc CM, Ma J, Taljaard M, Roth J, Scuccimarri R, Miettunen P (2015). Incident vertebral fractures and risk factors in the first three years following glucocorticoid initiation among pediatric patients with rheumatic disorders. J Bone Miner Res.

[11] Book C, Karlsson M, Akesson K, Jacobsson L (2008). Disease activity and disability but probably not glucocorticoid treatment predicts loss in bone mineral density in women with early rheumatoid arthritis. Scand J Rheumatol.

[12] Rouster-Stevens KA, Langman CB, Price HE, Seshadri R, Shore RM, Abbott K, Pachman LM (2007). RANKL:osteoprotegerin ratio and bone mineral density in children with untreated juvenile dermatomyositis. Arthritis Rheum.

[13] de Andrade DC, de Magalhães Souza SC, de Carvalho JF (2012). High frequency of osteoporosis and fractures in women with dermatomyositis/polymyositis. Rheumatol Int.

[14] Kanis JA, Johnell O, Oden A, Johansson H, McCloskey E (2008). FRAX and the assessment of fracture probability in men and women from the UK. Osteoporos Int.

[15] Bohan A, Peter JB (1975). Polymyositis and dermatomyositis (first of two parts). N Engl J Med.

[16] Aletaha D, Neogi T, Silman AJ, Funovits J, Felson DT, Bingham CO (2010). 2010 rheumatoid arthritis classification criteria: an American College of Rheumatology/European league against rheumatism collaborative initiative. Ann Rheum Dis.

[17] Consensus development conference: Diagnosis, prophylaxis, and treatment of osteoporosis. Am J Med. 1993;94:646–50.. 10.1016/0002-9343(93)90218-e 8506892

[18] John A (2011). Kanis: Fracture Risk Assessment Tool.

[19] Vereckei E, Susanszky E, Kopp M, Ratko I, Czimbalmos A, Nagy Z (2013). Psychosocial, educational, and somatic factors in chronic nonspecific low back pain. Rheumatol Int.

[20] Fries JF (1991). The hierarchy of quality-of-life assessment, the health assessment questionnaire (HAQ), and issues mandating development of a toxicity index. Control Clin Trials.

[21] Grados F, Fechtenbaum J, Flipon E, Kolta S, Roux C, Fardellone P (2009). Radiographic methods for evaluating osteoporotic vertebral fractures. Joint Bone Spine.

[22] Kanis JA, Johansson H, Oden A, McCloskey EV (2011). Guidance for the adjustment of FRAX according to the dose of glucocorticoids. Osteoporos Int.

[23] Gupta L, Lawrence A, Edavalath S, Misra R (2018). Prevalence and predictors of asymptomatic vertebral fractures in inflammatory myositis. Int J Rheum Dis.

